# Genomic sequence data and single nucleotide polymorphism genotyping of *Bacillus anthracis* strains isolated from animal anthrax outbreaks in Northern Cape Province, South Africa

**DOI:** 10.1016/j.dib.2019.105040

**Published:** 2019-12-24

**Authors:** Kgaugelo Edward Lekota, Ayesha Hassim, Henriette van Heerden

**Affiliations:** a University of Pretoria, Faculty of Veterinary Science, Department of Veterinary Tropical Diseases, Onderstepoort, 0110, South Africa; b College of Agriculture and Environmental Sciences, University of South Africa, Florida Campus, Florida, 1710, South Africa

**Keywords:** *Bacillus anthracis*, Whole genome sequencing (WGS), Canonical single nucleotide polymorphism (canSNP)

## Abstract

This report presents genomic data on sequence reads and draft genomes of *Bacillus anthracis* isolates from anthrax outbreaks in animals in an endemic region of South Africa as well as genotyping of the strains using canonical single nucleotide polymorphisms (canSNPs). It is derived from an article entitle “Phylogenomic structure of *B. anthracis* strains in the Northern Cape Province, South Africa revealed novel single nucleotide polymorphisms”. Whole genome sequencing (WGS) of twenty-three *B. anthracis* strains isolated during 1998 and 2009 anthrax outbreaks in the Northern Cape Province (NCP), as well as a strain from Botswana (6102_6B) and one from Namibia-South Africa transfrontier conservation area (Sendlingsdrift, 6461_SP2) were obtained using both the HiSeq 2500 and MiSeq Illumina platforms. Mismatch amplification mutation assay (melt-MAMA) qPCR were used to identify the canSNP genotypes within the global population of *B. anthracis*. DNA sequencing data is available at NCBI Sequence Read Archive and GenBank database under accession N0. PRJNA580142 and PRJNA510736 respectively. A phylogenetic tree and CanSNP typing profiles of the isolates are presented within this article.

**Table:** Specifications Table

Subject	Microbial genomics
Specific subject area	Comparative microbial genomics of *B. anthracis* strains for evolution and genetic diversity using single nucleotide polymorphisms (SNPs)
Type of data	Sequence files, Table, figure
How data were acquired	DNA extraction was performed on pure cultures using DNA Mini kit (Qiagen) purification kit. High-throughput DNA sequencing using Illumina HiSeq 2500 and MiSeq Sequencing system. De novo assemblies was performed using CLC-Genomic workbench version 11.1. Assembled genomes were annotated using NCBI Prokaryotic Genome Annotation Pipeline version 4.7. SNP genotyping Can SNP typing scheme was performed on LightCycler™ 96 (Roche Applied Science). MEGA version 7 was used to generate phylogenetic tree.
Data format	Raw and analysed data of whole genome sequences (Fastq and fasta)
Parameters for data collection	Samples were collected from animals that died of anthrax. Isolated pure cultures from sheep blood agar were used for DNA extractions, genotyping and sequencing.
Description of data collection	Pure culture isolates were identified using classical bacteriological methods including penicillin and bacteriophage sensitivity. DNA samples of these isolates were verified using *B. anthracis* plasmid and chromosomal gene targets using real- time PCR. Trimmed sequence reads were used for *de novo* assembly.
Data source location	University of Pretoria, Department of Veterinary and Tropical Diseases, Pretoria, South Africa
Data accessibility	The sequenced data were deposited to Sequence Read Archive (SRA) and GenBank in National Center for Biotechnology Information (NCBI). Accession numbers are included in this manuscript in a table format. BioProject numbers: PRJNA510736 (https://www.ncbi.nlm.nih.gov/bioproject/PRJNA510736) PRJNA580142 (https://www.ncbi.nlm.nih.gov/sra/PRJNA580142) With the article
Related research article	Lekota KE, Bezuidt OKI, Mafofo J, Rees J, Muchayeyi FC, Madoroba E, van Heerden H. Whole genome sequencing and identification of *Bacillus endophyticus* and *B. anthracis* isolated from anthrax outbreaks in South Africa. BMC Microbiology (2018) 18:67. doi: 10.1186/s12866-018-1205-9 [1]

**Value of the Data** • The data sheds light of draft genomes and genetic diversity of *B. anthracis* strains from Northern Cape Province from two anthrax outbreaks during 1998 and 2009 in South Africa. • The data serve as a benchmark for other researchers to determine the evolution and genetic diversity of *B. anthracis* globally. • The data could be used to determine the relationship between *B. anthracis* strains from South Africa and other areas and to expand the canSNP typing scheme using melt-MAMA. • The data might enable trace-back in and between anthrax cases/outbreaks, especially within the context of southern Africa.

## 1 Data description

**Fig. 1:**
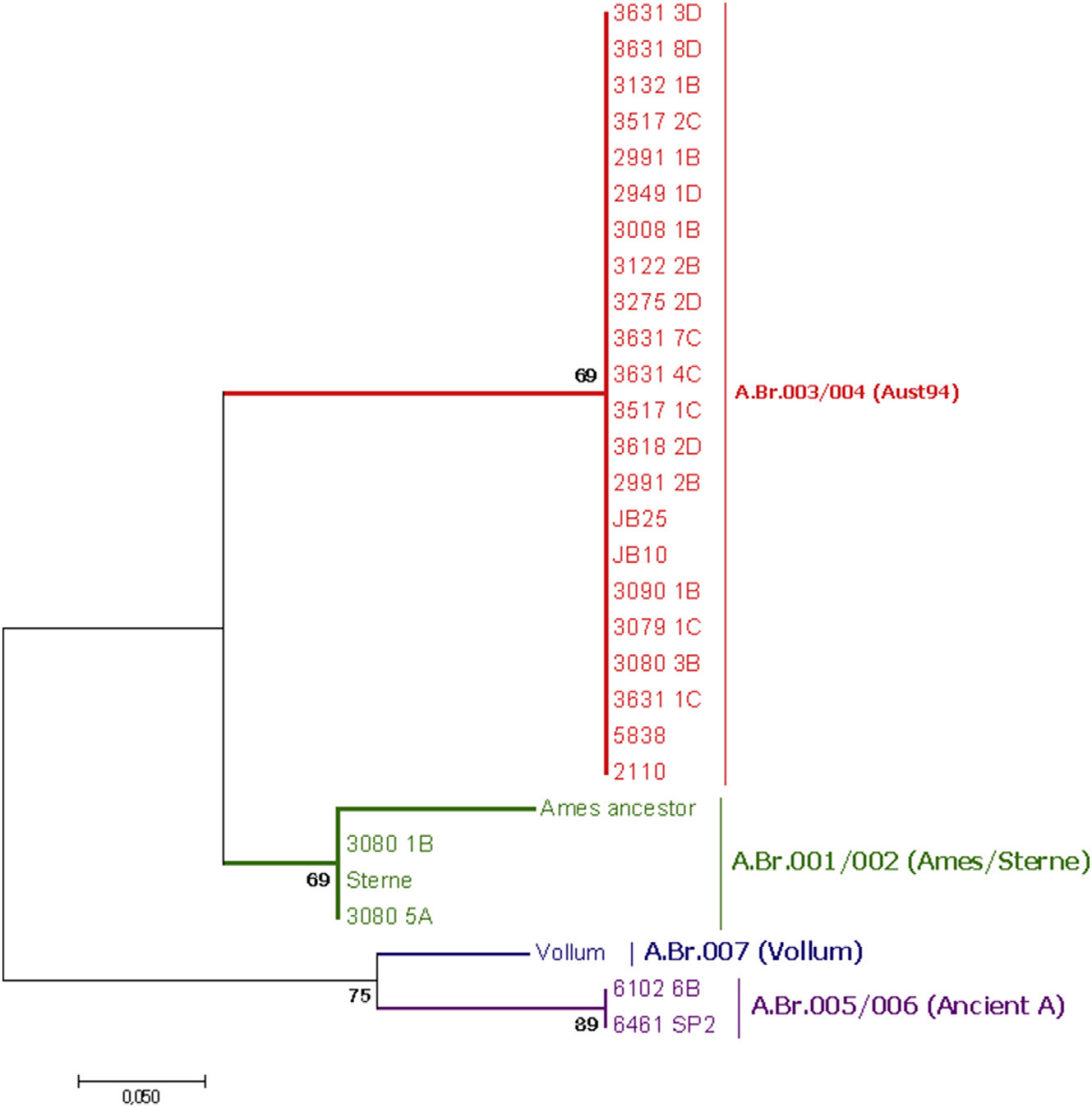
Maximum likelihood phylogeny of the major canonical single nucleotide polymorphism (canSNP) groups for the 26 *B. anthracis* strains as well as *B. anthracis* Ames ancestor, Vollum and Sterne control sequences. Most of the *B. anthracis* strains (n = 21) grouped in the canSNP A.Br 003/004 (Aust94) clade (red), while two strains, 3080_1B and 3080_5A, isolated from bovine grouped in A.Br.001/002 (Sterne) group (green) and isolates from Botswana (6102_6B) and Sendlingsdrift (6461_SP2) grouped in the A.Br.005/006 (Ancient a) group (purple).

We present the genomic data and analysis of whole genome sequences of *B. anthracis* strains isolated from animals anthrax outbreaks in Northern Cape Province. Sequence reads (in fastq format) and assembled genomes (in fasta format) were deposited at NCBI SRA and GenBank database under project accession No. PRJNA580142 and PRJNA510736 respectively. The information on the sample collection with accession numbers, SNP genotyping and genome assemblies is represented in Table 1, Table 2, Table 3 respectively. Isolates were also grouped using canonical SNPs (Table 4) typing scheme [2] used for phylogenetic branches (Fig. 1).

**Table 1:** Whole genome sequences of *Bacillus anthracis* strains collection with their accession numbers submitted to GenBank and Sequence Reads Achieve (SRA).

Strain name	Host	Collection date	Location	Accession number	Sequence coverage
2949_1D	Ovine	10-May-2009	South Africa: Northern Cape Province	RXZW00000000	145
2991_1B	Ovine	10-May-2009	South Africa: Northern Cape Province	RXZV00000000	199
3008_1B	Bovine	10-May-2009	South Africa: Northern Cape Province	RXZU00000000	155
3122_2B	*Oryx gazella*	10-May-2009	South Africa: Northern Cape Province	RXZT00000000	168
3132_1B	*Tragelaphus strepsiceros*	10-May-2009	South Africa: Northern Cape Province	RXZS00000000	201
3275_2D	Soil	10-May-2009	South Africa: Northern Cape Province	RXZR00000000	267
3517_1C	*Tragelaphus strepsiceros*	10-May-2009	South Africa: Northern Cape Province	RXZQ00000000	166
3517_2C	*Tragelaphus strepsiceros*	10-May-2009	South Africa: Northern Cape Province	RXZP00000000	137
3631_4C	*Tragelaphus strepsiceros*	10-May-2009	South Africa: Northern Cape Province	RXZO00000000	187
3631_3D	*Tragelaphus strepsiceros*	10-May-2009	South Africa: Northern Cape Province	RXZN00000000	189
3631_8D	*Tragelaphus strepsiceros*	10-May-2009	South Africa: Northern Cape Province	RXZM00000000	300
2110	*Ovis aries*	1998	South Africa: Northern Cape Province	RXZL00000000	38
JB10	*Equus burchellii quagga*	2009	South Africa: Northern Cape Province	RXZK00000000	60
JB25	*Tragelaphus strepsiceros*	2009	South Africa: Northern Cape Province	SDEF00000000	80
3618_2D	*Tragelaphus strepsiceros*	10-May-2009	South Africa: Northern Cape Province	RXZJ00000000	178
6461_SP2	*Capra aegagrus*	2009	South Africa: Northern Cape Province	SRP227303; SAMN13151840; SRR10357978	20
6102_6B	*Loxodonta*	2009	Botswana	SRP227303; SAMN13151841; SRR10357979	21
3631_7C	Soil	2009	South Africa: Northern Cape Province	SRP227303; SAMN13151842; SRR10357981	24
5838	*Alcelaphus buselaphus*	1998	South Africa: Northern Cape Province	SRP227303; SAMN13151843; SRR10357980	17
2991_2B	Ovine	2009	South Africa: Northern Cape Province	SRP227303; SAMN13151844; SRR10357985	19
3080_3B	Bovine	2009	South Africa: Northern Cape Province	SRP227303; SAMN13151845; SRR10357983	17
3079_1C	*Oryx gazella*	2009	South Africa: Northern Cape Province	SRP227303; SAMN13151846; SRR10357984	25
3080_5A	Bovine	2009	South Africa: Northern Cape Province	SRP227303; SAMN13151847; SRR10357982	26
3080_1B	Bovine	2009	South Africa: Northern Cape Province	SRP227303; SAMN13151848; SRR10357977	12
3090_1B	Unknown	2009	South Africa: Northern Cape Province	SRP228283; SAMN10614343; SRR10390628	26

**Table 2:** Canonical SNPs used for genotyping of *B. anthracis* strains. SNP are in relation to *B. anthracis* Ames ancestor chromosome (NC_007530.2).

B. anthracis Strains	SNP-branch	A.Br.006	A.Br.007	A.Br.008	A.Br.005	A.Br.004	A.Br.003	A.Br.002	A.Br.001	A.Br.009	A.Br.011	A.Br.014	A.Br.013

	Ancestral Template SNP	C	A	A	A	T	T	C	T	A	G	T	A
	Derived Template SNP	A	G	C	G	C	C	T	C	G	A	C	G
Ames ancestor	A.Br.001 (Ames)	A	A	A	G	C	C	T	C	A	G	T	A
Sterne	A.Br.002 (Sterne)	A	A	A	G	C	C	T	T	A	G	T	A
3080_5A	A.Br.002 (Sterne)	A	A	A	G	C	C	T	T	A	G	T	A
3080_1B	A.Br.002 (Sterne)	A	A	A	G	C	C	T	T	A	G	T	A
6102_6B	A.Br.005/006 (Ancient A)	A	A	A	A	T	T	C	T	A	G	T	A
6461_SP2	A.Br.005/006 (Ancient A)	A	A	A	A	T	T	C	T	A	G	T	A
2110	A.Br.003/004 (A.Br.101)	A	A	A	G	C	C	C	T	A	G	C	A
5838	A.Br.003/004 (A.Br.101)	A	A	A	G	C	C	C	T	A	G	C	A
3631_1C	A.Br.003/004 (A.Br.101)	A	A	A	G	C	C	C	T	A	G	C	A
3080_3B	A.Br.003/004 (A.Br.101)	A	A	A	G	C	C	C	T	A	G	C	A
3079_1C	A.Br.003/004 (A.Br.101)	A	A	A	G	C	C	C	T	A	G	C	A
3090_1B	A.Br.003/004 (A.Br.101)	A	A	A	G	C	C	C	T	A	G	C	A
JB10/NC14	A.Br.003/004 (A.Br.101)	A	A	A	G	C	C	C	T	A	G	C	A
JB25/NC_29	A.Br.003/004 (A.Br.101)	A	A	A	G	C	C	C	T	A	G	C	A
2991_2B	A.Br.003/004 (A.Br.101)	A	A	A	G	C	C	C	T	A	G	C	A
3618_2D	A.Br.003/004 (A.Br.101)	A	A	A	G	C	C	C	T	A	G	C	A
3517_1C	A.Br.003/004 (A.Br.101)	A	A	A	G	C	C	C	T	A	G	C	A
3631_4C	A.Br.003/004 (A.Br.101)	A	A	A	G	C	C	C	T	A	G	C	A
3631_7C	A.Br.003/004 (A.Br.101)	A	A	A	G	C	C	C	T	A	G	C	A
3275_2D	A.Br.003/004 (A.Br.101)	A	A	A	G	C	C	C	T	A	G	C	A
3122_2B	A.Br.003/004 (A.Br.101)	A	A	A	G	C	C	C	T	A	G	C	A
3008_1B	A.Br.003/004 (A.Br.101)	A	A	A	G	C	C	C	T	A	G	C	A
2949_1D	A.Br.003/004 (A.Br.101)	A	A	A	G	C	C	C	T	A	G	C	A
2991_1B	A.Br.003/004 (A.Br.101)	A	A	A	G	C	C	C	T	A	G	C	A
3517_2C	A.Br.003/004 (A.Br.101)	A	A	A	G	C	C	C	T	A	G	C	A
3132_1B	A.Br.003/004 (A.Br.101)	A	A	A	G	C	C	C	T	A	G	C	A
3631_3D	A.Br.003/004 (A,Br.101)	A	A	A	G	C	C	C	T	A	G	C	A
3631_8D	A.Br.003/004 (A.Br.101)	A	A	A	G	C	C	C	T	A	G	C	A
Aust94	A.Br.003/004 (Aust94)	A	A	A	G	C	C	C	T	A	G	C	G
Vollum	A.Br.007 (Vollum)	A	G	A	G	T	T	C	T	A	G	T	A

**Table 3:** Genomic features of the *de novo* assemblies *B. anthracis* strains (n = 15) using CLC Genomic workbench.

Strain name	Sequence coverage	Number of contigs	N50	Minimum contig size (bp)	Maximum contig size (bp)	GC content	Genome Size	Total coding sequences (CDSs)	Total number of RNAs
2949_1D	145	441	28 406	423	125 072	35.1	5 147 319	5 764	65
2991_1B	199	378	38 630	316	185 192	35.1	5 395 612	5 736	54
3008_1B	155	442	34 402	406	226 189	35.1	5 418 987	5 763	63
3122_2B	168	431	34 419	361	175 230	35.1	5 401 847	5 740	54
3132_1B	201	170	74 712	146	335 422	35.1	5 350 330	5 611	97
3275_2D	267	751	14 738	509	89 998	35.1	5 352 180	5 463	59
3517_1C	166	121	203 477	354	343375	35.1	5 416 293	5 692	68
3517_2C	137	1194	9 613	352	55 932	35.1	5 265 628	5 869	37
3631_4C	187	385	35 768	418	177 852	35.1	5 402 081	5 718	68
3631_3D	189	513	22 221	415	108 007	35.1	4 654 382	5 766	52
3631_8D	300	882	14 279	401	98 835	35.1	5 252 949	5 717	68
2110	38	856	7 046	517	77 020	35.0	3 843 425	5 906	74
JB10	60	1856	6 493	153	50 654	35.1	5 180 538	5 861	34
JB25	80	136	91 967	519	646 630	35.1	5 422 668	5 695	88
3618_2D	178	72	154 041	2803	489 427	35.1	5 417 873	5 674	62

**Table 4:** Melt-MAMA primers targeting canonical SNPs of the existing Birdsell et al. (2012) primers used in this study for the phylogenetic branches.

Assay name	aReference genome position	Derived MAMA 5′-3′	Ancestral MAMA 5′-3′	Common reverse 5′-3′	Annealing Temperature (°C)
Existing primers by Birdsell et al., 2012					
A.Br.001	182 106	cggggcggggcggggcgggcAGAAGGAGCAAGTAATGTTATAGGTTTAaGT	GGAGCAAGTAATGTTATAGGTTTAcGC	ACCTAAAATCGATAAAGCGACTGC	55
A.Br.002	947 760	cggggcggggcggggcgggcAGAAGGAGCAAGTAATGTTATAGGTTTAaGT	GGAGCAAGTAATGTTATAGGTTTAcGC	ACCTAAAATCGATAAAGCGACTGC	55
A.Br.003	1 493 280	cggggcggggcggggcgggcAATTTAGATTTTCGTGTCGAATTAtGC	AATTTAGATTTTCGTGTCGAATTAgGT	TGTATAAAAACCTCCTTTTTCTACCTCAA	55
A.Br.004	3 600 786	cggggcggggcggggcgggcCGCCGTCATACTTTGGAAaGC	CGCCGTCATACTTTGGAAcGT	GAATTGGTGGAGCTATGGAAGGATTA	60
A.Br.005	3 842 864	cggggcggggcggggcgggcGAAAGATATATAAAAATGTTTTTTTATTTCGTtTG	GAAAGATATATAAAAATGTTTTTTTATTTCGTcTA	GCTGCGTTTAGTTATGCAAATC	55
A.Br.006	162 509	cggggcggggcggggcgggcAATATGTTGTTGATCATTCCATCGCtTA	TATGTTGTTGATCATTCCATCGCgTC	TAGCGTTTTTAAGTTCATCATACCCATGC	55
A.Br.007	266439	cggggcggggcggggcgggcACAAGGTGGTAGTATTCGAGCTGAtTG	AATTACAAGGTGGTAGTATTCGAGCTGAcTA	CGAGACGATAAACTGAATAATACCATCCT	62.5
A.Br.008	3947375	cggggcggggcggggcgggcGTTACAAATATACGTTTAACAAGCcGC	AAAAGTTACAAATATACGTTTAACAAGCtGA	CTACGCTATACGTTTTAGATGGAGATAATTC	55
A.Br.009	2589947	cggggcggggcggggcgggcCCACTGTTTTTGAACGGCTcTG	GCCACTGTTTTTGAACGGCTaTA	TTTTAGGTATATTAACTGCGGATGATGC	60
A.Br.011	1455402	cggggcggggcggggcgggcCATAAAAGAAATCGGTACAATAGAAtAG	CATAAAAGAAATCGGTACAATAGAAcAA	TCGGATATGATACCGATACCTTCTTATC	55
A.Br.014	5078168	ggggcggggcggggcggggcggggcAATGGTAAATTGTAATGTTGAGCTtC	AATGGTAAATTGTAATGTTGAGCTgT	TTTTTACTAAAAAATTACTTTTTTTGAAAA	57
A.Br.013	2465446	ggggcggggcggggcggggcggggcTTGTAAAAATTCTATGTGAATCACATtG	TTGTAAAAATTCTATGTGAATCACATcA	TTATCCACCTTCTTATAATTATTTATTACTAT	57

GC-clamp (cggggcggggcggggcgggc).

a *Bacillus anthracis* Ames ancestor reference genome (NC_007530.2).

## 2 Experimental design, materials, and methods

### 2.1 Diagnostic real-time PCR for chromosomal and plasmids markers of *B. anthracis*

The identification of *B. anthracis* isolates was performed as described by WHO [3]. The 20 μl PCR reaction consisted of 10 μl of FastStart Essential master mix (Roche Applied Science), 0.5 μM of each primer, 0.2 μM of probe for each chromosomal and plasmid target pairs with fluorescein on the one and LCRed640 on the other (Tib MolBiol GmbH, Germany) and 2.5 μl of template DNA. The PCR conditions on a LightCycler™ Nano (Roche Applied Science) were used as described in WHO [3]. The PCR conditions on a LightCycler™ Nano (Roche Applied Science) consisted of an initial cycle at 95 °C for 10 minutes, slope at 20 °C/second, followed by 40 cycles of 95 °C for 10 seconds; 57 °C for 20 seconds; 72 °C for 30 seconds, slope 20 °C/second with one single signal acquisition at the end of annealing cycle. Denaturation at 95 °C for 3 seconds with a slope 20 °C/second; 40 °C for 30 seconds, slope 20 °C/second; 80 °C for 3 seconds at a slope of 0.1 °C/second with continuous acquisition of the signal. Cooling to 40 °C for 30 seconds, slope 20 °C/second.

### 2.2 Genotyping of *B. anthracis* strains using Melt-MAMA assays

Melt-MAMA assays of the canSNP markers were used to amplify the DNA of the NCP *B. anthracis* strains. The panel included 12 canSNPs that were used for the grouping of the *B. anthracis* strains (n = 26) using existing Melt-MAMA primers (Table 4) derived and ancestral controls were created as described by Birdsell et al. [2]. The reaction included 2.5 μl DNA diluted in 1× FastStart DNA Green Master (Roche Applied Science) with an ancestral forward and a derived forward SNP target primer (GC-clamp: no-GC-clamp) and a common reverse primer (Inqaba Biotec™) (Table 2) with a starting concentration of 0.2 μM depending on the ratio indicated which allowed for separation of melt peaks by at least 5 °C. Thermocycling parameters on the LightCycler™ 96 (Roche Applied Science) were 95 °C for 10 minutes, followed by 35 cycles at 95 °C for 15 seconds and 55 °C-60 °C (oligonucleotide dependent for 1 minute) for 35 cycles. End-point PCR amplicons were subjected to melt analysis using a dissociation protocol comprising of 95 °C for 15 seconds, followed by incremental temperature ramping (0.1 °C) from 60 °C to 95 °C. SYBR Green fluorescence intensity was measured at 530 nm at each ramp interval and plotted against temperature and observed as the separate melt peaks for each SNP. Controls included in every run were DNA from *B. anthracis* Ames, Vollum and Sterne 34F_2_ strains. Phylogenetic relationships between 26 *B. anthracis* strains were determined in the MEGA version 7 [4] using the maximum likelihood method based on the Tamura three-parameter model. The tree was generated with a bootstrap replication value of 500.

### 2.3 High-throughput sequencing and bioinformatics analysis

The DNA samples that were extracted from *B. anthracis* were subjected to library preparation by using the Nextera XT DNA Sample Prep kit (Illumina-compatible, Epicentre Biotechnology). Different sequence reads of *B. anthracis* genomes were generated on HiSeq 2500 and MiSeq instruments platforms. Clusters were generated on the flow cell using HiSeq Paired-End Cluster Generation kit (Ilumina, USA) for the HiSeq 2500 platform. Sequencing of paired end libraries were performed on the Illumina MiSeq and HiSeq 2500 sequencer using the 200-cycle SBS (sequencing by synthesis) sequencing v3 kit (Illumina, USA) and HiSeq Sequencing Kit (200 cycles) (Illumina, USA) respectively. Quality of the genome sequenced reads were assessed using FastQC software 0:10.1 [5]. Trimommatic version 0.33 [6] was used to remove the sequenced adapter, and ambiguous nucleotide reads. *De novo* assemblies of the paired end reads were performed using CLC Genomics Workbench version 11.1 (CLC, Denmark). The assembled contigs were ordered by Mauve tool version 2.3.1 [7] using *B. anthracis* Ames ancestor (GenBank accession numbers NC_007530.2, NC_007322.2 and NC_007323.3) in order to assess the accuracy and efficiency of the contigs. All trimmed sequence reads were also mapped to the reference using Burrows-Wheeler Aligner (BWA) version 0.7.12 [8] to determine *B. anthracis* replicons i.e. chromosome and the two plasmids. Assembled genomes were annotated using the NCBI Prokaryotic Genome Annotation pipeline. Sequenced reads were deposited to NCBI under Sequence Reads Archive (SRA), and assembled genomes to GenBank.
